# Intraoperative Use of Methadone for Postoperative Pain Control in Bariatric Surgery: A Randomized, Double-Blind, Controlled Clinical Trial

**DOI:** 10.7759/cureus.77127

**Published:** 2025-01-08

**Authors:** Juliana M Freire, Silas Augusto G dos Santos, Raphael K Confessor de Sousa, Matheus S Nascimento, Heitor JS Medeiros, Rand R Martins, Pedro H De Medeiros Silva, Eudes P de Godoy, Wallace Da Silva

**Affiliations:** 1 Department of Anesthesiology, Hospital Universitário Onofre Lopes, Natal, BRA; 2 Department of Anesthesiology, Critical Care and Pain Medicine, Massachusetts General Hospital, Boston, USA; 3 Graduate Program of Pharmaceutical Assistance, Universidade Federal do Rio Grande do Norte, Natal, BRA; 4 Department of Anesthesiology, Universidade Federal do Rio Grande do Norte, Natal, BRA; 5 Department of Obesity Surgery Service and Related Diseases, Universidade Federal do Rio Grande do Norte, Natal, BRA; 6 Department of Anaesthesiology, Hospital Universitário Onofre Lopes, Natal, BRA

**Keywords:** anesthesia, bariatric surgery, methadone, obesity, postoperative pain

## Abstract

Introduction: This study aimed to assess postoperative pain in patients who received a single dose of methadone during the anesthetic-surgical procedure, as well as to evaluate adverse effects.

Methods: A randomized, double-blind, clinical trial with patients undergoing video laparoscopic bariatric surgery. Immediately after anesthesia induction, the methadone group (MG) received 10 mg of methadone diluted in 100 ml of 0.9% saline, and the Control group (CG) received only 100 ml of 0.9% saline (without methadone in this group). The assessment of pain was made using the visual analog scale (VAS), 10 minutes after extubation in the post-anesthesia care unit (PACU) and six, 12, and 24 hours after surgery. The presence of nausea and vomiting, respiratory depression, and the need for postoperative rescue opioids were analyzed.

Results: A total of 16 patients were allocated in the MG and 18 in the CG. The MG showed a lower average of pain scores on the VAS over 24 hours, with a significant difference in the first 10 minutes postoperatively. Furthermore, it showed a greater decrease in VAS pain scores over 24 hours (β = -1.984; p<0.001) compared to the CG. In the postoperative period, no significant difference was found between the groups regarding rescue morphine use within the first 24 hours (p=0,469), except during the PACU period, the rescue morphine use was higher in the CG (p=0,005). There was no significant difference regarding the presence of nausea and vomiting (p=0.372).

Conclusion: Our findings suggested that intravenous methadone single dose in laparoscopic bariatric surgery was safe, achieving better postoperative pain outcomes.

## Introduction

Laparoscopic bariatric surgery is recognized as the most effective intervention for treating morbid or complicated obesity. However, anesthesia for these patients presents unique challenges, notably in managing postoperative pain with minimal opioid-related side effects. Up to 50% of these patients report significant postoperative pain, which can adversely affect respiratory function, hemodynamics, risk of acute myocardial infarction, cardiac arrhythmias, mobility, intestinal function, wound healing, length of hospital stay, and overall patient satisfaction. Poor pain management can also lead to chronic pain, further diminishing patient well-being [1-4].

Typically, shorter-acting opioids like morphine or hydromorphone are administered through intermittent intravenous boluses for postoperative analgesia. This approach can result in fluctuating blood levels of opioids, leading to a spectrum of clinical outcomes ranging from inadequate pain relief to excessive sedation and respiratory depression [1-5]. Furthermore, using rescue opioids such as morphine can introduce complications including pruritus and urinary retention. Obese patients, in particular, are concerned about respiratory complications due to their altered breathing patterns and higher risk of respiratory issues [1-6].

Methadone, a long-acting opioid, offers stable plasma concentrations after a single dose, providing extended pain relief without the peaks and troughs associated with repeated administrations [5,7]. Beyond its role as a μ-receptor agonist, methadone antagonizes the N-methyl-D-aspartate (NMDA) receptor and inhibits the reuptake of serotonin and noradrenaline in the central nervous system. When given intravenously, methadone begins to take effect within 10-20 minutes and its duration of action ranges from four to 36 hours, influenced by the dose and genetic factors affecting liver metabolism. Intravenous doses of 20-30 mg can sustain pain relief for approximately 24-36 hours [7,8]. 

Anesthesiologists must navigate the challenge of mitigating postoperative pain with minimal risk of complications, all while considering the cost-benefit ratio. Evidence suggests that incorporating methadone into the anesthesia regimen can significantly reduce postoperative opioid consumption, lower pain scores, and enhance patient satisfaction [2].

This study aimed to assess postoperative pain in patients who received a single dose of methadone during the anesthetic-surgical procedure, as well as to evaluate adverse effects such as nausea and vomiting, respiratory depression, and the need for postoperative rescue opioids.

## Materials and methods

This was a randomized, double-blind, controlled clinical trial conducted at University Hospital Onofre Lopes (HUOL), Natal, Brazil, from October 2023 to December 2023. The study was approved by the Ethics Committee of HUOL (approval number: 52832821.2.0000.5292) on February 4, 2022. Subsequently, the trial was registered at the Brazilian Registry of Clinical Trials (ReBEC) under the identifier U-1111-1287-2054. Following that, written informed consent was obtained from all subjects participating in the trial.

Inclusion and exclusion criteria

The participants included in the study underwent video laparoscopic bariatric surgery performed by the attending surgeons and anesthesiologists at HUOL. The inclusion criteria were as follows: American Society of Anesthesiologists (ASA) class II or III patients, aged 18-65 years, scheduled for video laparoscopic bariatric surgery with six portals. The exclusion criteria comprised patient refusal, history of drug addiction, chronic opioid use (greater than three months), fibromyalgia or chronic pain (duration exceeding three months), QT interval prolongation, allergy to methadone or non-steroidal anti-inflammatory drugs (NSAIDs), creatinine clearance rate below 60 ml/min/1.73m², and body mass index (BMI) over 55 kg/m². 

Sample size

The sample size was estimated to detect differences of three units in the pain VAS scores between the groups at 24 hours post-extubation, also considering a standard deviation of four units with 80% power and an alpha error of 5% [7]. With an addition of 10% accounting for possible losses, a total of 32 participants in each group was reached. Upon achieving 50% of the anticipated sample size, an interim analysis was conducted, and it was decided to conclude data collection with 34 participants due to the significance of the findings. 

Randomization and blinding

Patients were allocated into two groups by an assistant anesthesiologist using simple randomization with sealed and numbered envelopes: (i) methadone group (MG) and (ii) control group (CG). Demographic data were collected. A separate assistant, blinded to other aspects of the study, prepared either a solution with 10 mg of methadone diluted in 100 ml of 0.9% saline or a control solution with 100 ml of 0.9% saline. The methadone dosage was determined to be between 0.1 to 0.2 mg/kg of ideal body weight (IBW), increased by 20%, calculated using Robinson's formula [9], which is currently considered the most accurate equation for determining IBW. This assistant did not participate in any other part of data collection, and patient information and allocation were kept in opaque sealed envelopes. The selected solution was administered after the induction of general anesthesia. The study adhered to the applicable Consolidated Standards of Reporting Trials (CONSORT) guidelines [10]. All other assistants and healthcare professionals involved in the study, including those conducting preoperative, intraoperative, and postoperative assessments, were blinded.

Intervention

All patients received standard monitoring, including continuous five-lead electrocardiogram, pulse oximetry, non-invasive blood pressure measurement, esophageal temperature monitoring, capnography, and gas analysis. Anesthesia was induced with propofol, sufentanil, and a neuromuscular blocker (NMB), either cisatracurium or rocuronium. Anesthesia maintenance consisted of sevoflurane at 1.8-2%. The induction doses and choice of NMB were at the discretion of the anesthesiologist. Titrated NMB doses were administered to ensure optimal surgical relaxation. Hypotension, defined as a reduction of 20% or more from the initial blood pressure, was managed with boluses of either metaraminol (0.5 mg) or ephedrine (5 mg), based on clinical indications. No neuraxial or peripheral blocks were performed. The MG received 10 mg of methadone intravenously, diluted in 100 ml of 0.9% saline solution, immediately after anesthesia induction. The CG received 100 ml of 0.9% saline intravenously, without any medication, after anesthesia induction.

Adjuvants were employed to achieve enhanced multimodal analgesia and mitigate postoperative nausea and vomiting (PONV) at the initiation of the procedure (administering dexamethasone 4 mg) and upon its conclusion (administering dipyrone 2 g, ondansetron 8 mg, and tenoxicam 40 mg). They could not receive other medications that interfere with pain control such as remifentanil, esketamine, dexmedetomidine, magnesium sulfate, and lidocaine. Before extubation, all patients were administered neostigmine 30 µg/kg and atropine 15 µg/kg if cisatracurium was utilized, or sugammadex 2 mg/kg if rocuronium was utilized, guided by a train of four monitors. 

If the patient experienced pain in the post-anesthesia care unit (PACU), intravenous morphine was administered, in a titrated dose of 1 mg every 1 mg, every five minutes. If sustained desaturation or hypoventilation with less than 90% oxygen saturation, patients received supplementary oxygen, 2 liters per minute, by nasal catheter. In the ward, the patients received a fixed dose of dipyrone 1 g IV every six hours, tenoxicam 40 mg once a day, and nalbuphine 10 mg IV up to every six hours was prescribed for pain relief, as needed. For data analysis, a dose equivalent of nalbuphine to morphine was performed. 

Pain was assessed using the Visual Analog Scale (VAS) on a 0-10 scale, where 0 represents no pain and 10 represents the worst possible pain. Side effects were also evaluated 10 minutes after arrival in the PACU. Patients were transferred to the general ward once an Aldrete-Kroulik score greater than 9 was achieved.

Outcomes

The primary outcomes were pain assessment using VAS 10 minutes after arrival in the PACU and at intervals of six, 12, and 24 hours post-surgery. The secondary outcomes were the presence of nausea and vomiting, the need for supplemental oxygen post-PACU discharge, respiratory depression, and opioid consumption in the first 24 hours after surgery.

Statistical analysis

The sample size of 17 participants in each group was estimated to detect differences of four units in the values ​​obtained on the VAS between the groups during a period of 12 hours after extubation, also considering a standard deviation of four units with 80% power and alpha error of 5% [7]. Baseline characteristics were reported as mean and standard deviation for continuous variables and frequency distribution for categorical ones. Data normality was assessed using the Shapiro-Wilk test. Pre-intervention group differences were evaluated using the Student's T-test or Wilcoxon test for continuous variables, and Pearson's chi-squared or Fisher's exact test for categorical variables. A random effects linear model compared VAS scale variations between the methadone and control groups, with VAS score as the dependent variable and time (0, 6, 12, and 24 hours post-surgery) as the fixed effect independent variable. Intraindividual variability, age, female gender, and BMI were included as random components in the model. In a secondary analysis, mean pain scores between groups in the first 10 minutes post-surgery were compared using the Student's T-test. A p-value < 0.05 was deemed significant. Sample calculation and statistical analyses were conducted using Stata Statistical Software: Version 18 (2017; StataCorp LLC, College Station, Texas, United States).

## Results

Study participants and demographics

**Figure 1:**
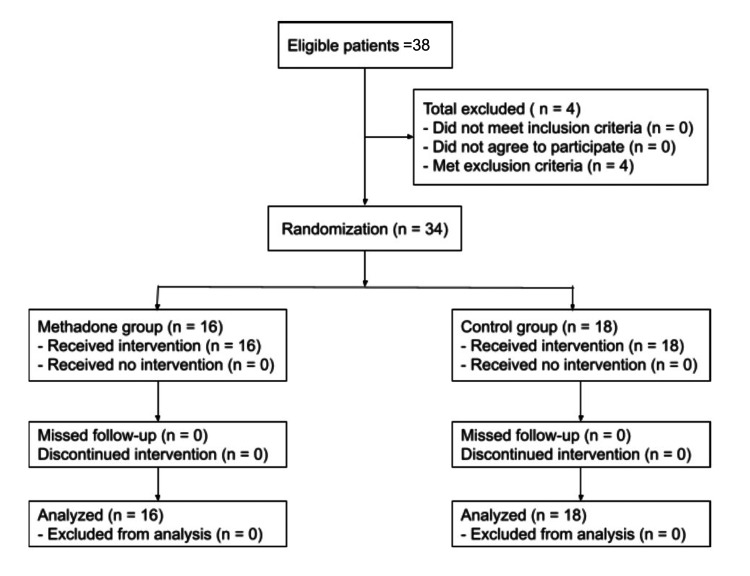
CONSORT flowchart showing the recruitment of study patients. CONSORT: Consolidated Standards of Reporting Trials

A total of 38 patients were deemed eligible for the study. Four patients met the exclusion criteria, leaving 34 patients for analysis: 16 in the MG and 18 in the CG (Figure 1). Both groups were comparable in terms of demographic characteristics. Ages ranged from 23 to 62 years, with an average of 40.9 (SD 12.5) years in the CG and 44.3 (SD 7.4) years in the MG. The most commonly performed surgery in both groups was sleeve gastrectomy (Table 1).

**Table 1:** Demographic characterization of the groups p-value < 0.05 was considered significant; Wilcoxon test for continuous variables and Fisher's exact test for categorical variables.

Characteristics	Control Group (n = 18)	Methadone group (n = 16)	P-value
Age (years), mean±SD	40.9±12.5	44.3±7.4	0.346
Female gender, n (%)	13 (72.2)	15 (93.8)	0.100
IMC (Kg/m*^2^)*, mean±SD	44.8±5.3	42.1±7.1	0.217
Type of surgery, n (%)			
Sleeve	10 (55.6)	8 (50.0)	0.841
Sleeve + Bipartition	2 (11.1)	2 (12.5)	
Second Split Time	2 (11.1)	1 (6.3)	
Bypass	2 (11.1)	2 (12.5)	
Bipartition	1 (5.6)	0 (0.0)	
Conversion from sleeve to bypass	0 (0.0)	1 (6.3)	
Vertical gastrectomy	1 (5.6)	1 (6.3)	
Sleeve + Bypass	0 (0.0)	1 (6.3)	

Intraoperative management

During the intraoperative period, both groups received similar management with no significant differences in the doses of propofol (p = 0.277), neuromuscular blockers (cisatracurium p = 0.582 or rocuronium p = 0.352), sufentanil (p = 0.551), or anesthesia duration (p = 0.790) (Table 2).

**Table 2:** Anesthetic management and postoperative data p-value < 0.05 was considered significant; Wilcoxon test for continuous variables and Fisher's exact test for categorical variables. PACU: post-anesthesia care unit

Characteristics	Control Group (n=18)		Methadone Group (n=16)	p-value
Sufentanil in ug, mean±SD	38.6±20.9		43.1±22.7	0.551
Propofol in mg, mean±SD	194.4±34.0		180.0±42.1	0.277
Neuromuscular blockers in mg, mean±SD				
Rocuronium	95.5±23.8		107.3±33.5	0.352
Cisatracurium	18.0±3.8		16.0 (8.2)	0.582
Morphine Rescue at first 24 hours, mean±SD	3.4±3.0		2.4±2.5	0.469
Morphine Rescue at PACU, n (%)	9 (50.0)		1 (6.3)	0.005
Morphine Rescue after discharge from PACU, mean±SD	1.7±0.6		2.5±1.0	0.259
Nausea or vomit episode, n (%)	11 (61.1)		7 (43.8)	0.311
Supplementary O2	0		0	
Duration of surgery in hours, mean±SD	4.3±1.1		4.4±1.3	0.790

Primary Outcome: Pain Scores

**Figure 2:**
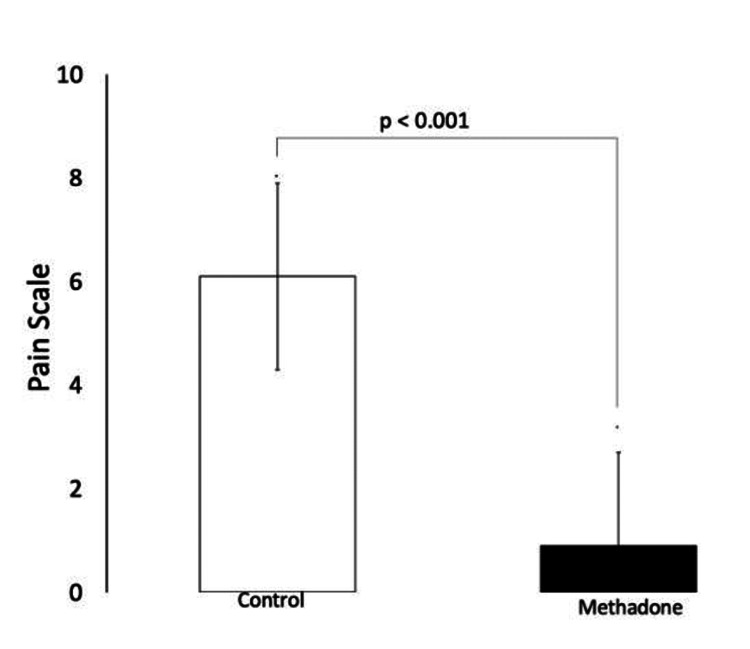
Pain scores 10 minutes after PACU arrival, assessed by VAS. p-value < 0.05 was considered significant (Wilcoxon test) PACU: post-anesthesia care unit; VAS: Visual Analog Scale

**Figure 3:**
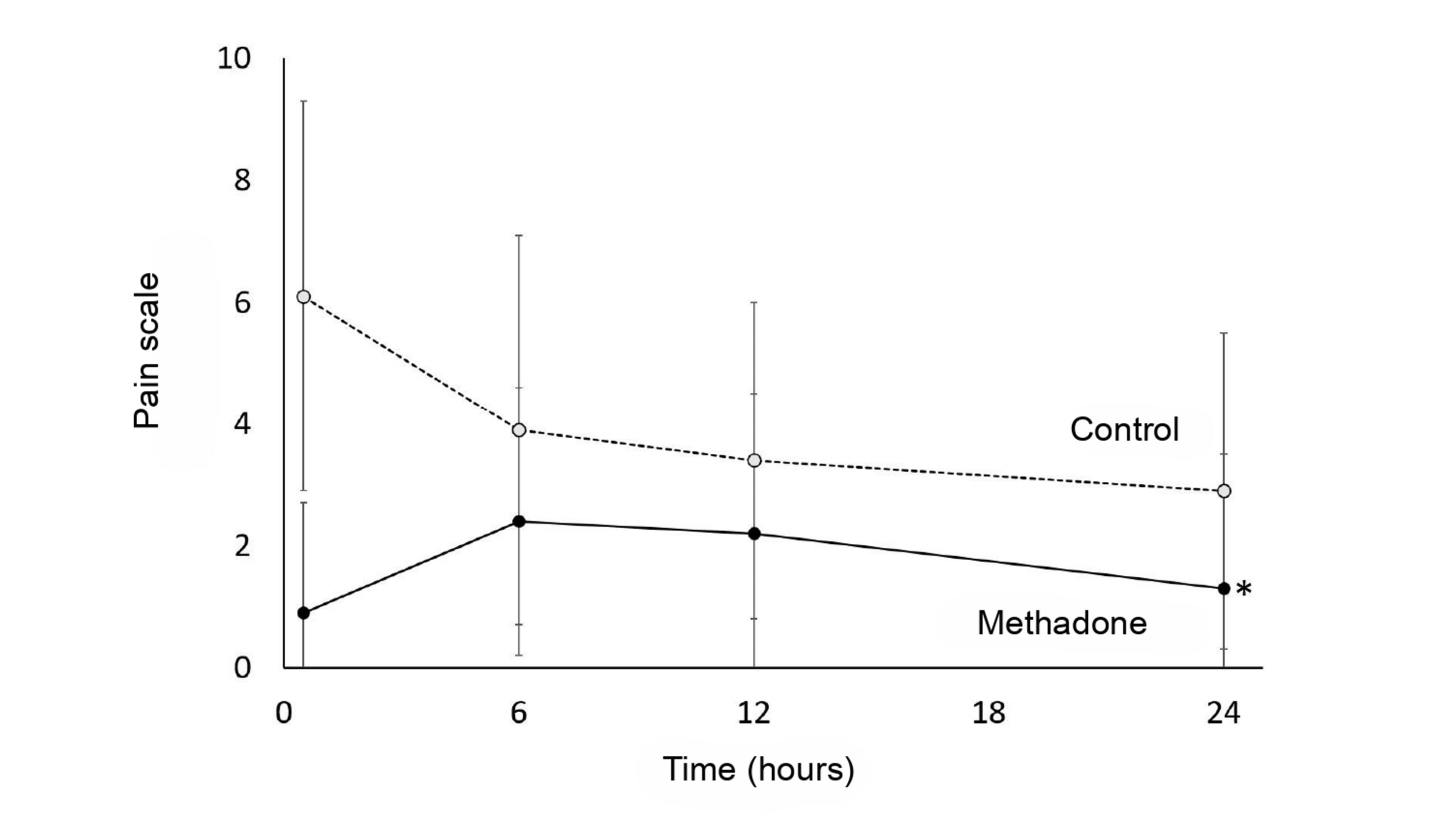
Mixed effects linear regression model showing the variation of the pain scale at 24 hours between the control and methadone groups. Note: The model was adjusted for sex and BMI. * p-value < 0.05.

Regarding the primary outcome, Table 3 shows each group's mean and SD of pain scores. When analyzing the score curve over 24 hours, the MG demonstrated a lower average pain score on the VAS, with a significant difference in the first 10 minutes after PACU arrival (Figure 2). Although the linear regression model indicated a general decrease in pain scores over time for all patients (β = -0.061; p = 0.001), the MG showed a more substantial and statistically significant decrease (β = -1.984; p < 0.05) compared to the CG (Figure 3 and Table 4).

**Table 3:** Pain scale values over time in the control and methadone groups. PACU: post-anesthesia care unit

Time	Pain Scale Values	
	Control Group (n=18), mean±SD	Methadone Group (n=16), mean±SD
10 minutes after arrival to PACU	7.7 ± 2.2	0.6 ± 1.3
Postoperative 6 hours	5.2 ± 3.1	2.4 ± 2.4
Postoperative 12 hours	4.5 ± 2.3	2.6 ± 2.4
Postoperative 24 hours	3.8 ± 2.5	1.3 ± 2.4

**Table 4:** Variables used in linear regression and statistical analysis. p-value < 0.05 was considered significant, mixed effects linear regression model

Variable	𝝱	SE	p-value	95% CI (lower limit, upper limit)	
Methadone usage	-1.984	0.734	0.007	-3.424	-0.546
Time (hours)	-0.061	0.018	0.001	-0.096	-0.027
Age (years)	-0.061	0.034	0.078	-0.128	0.007
Female sex	-0.382	0.945	0.686	-2.234	1.470
BMI (Km/m^2^)	0.029	0.057	0.612	-0.082	0.140
Intercept	7.974	2.124	<0.001	3.810	12.137

Postoperative Outcomes

In the postoperative period, there was no significant difference between the groups regarding rescue morphine use within the first 24 hours (p = 0.469). However, during the PACU period, the use of rescue morphine was significantly higher in the CG compared to the MG (p = 0.005). During this period, only one patient in the MG required 5 mg of rescue morphine, whereas nine patients in the CG required morphine for pain control. The rescue morphine doses in the CG ranged from 4 to 10 mg, with a mean dose of 6.4 mg (SD 2.7).

There was no significant difference between the groups in terms of the occurrence of nausea and vomiting (p = 0.311). Additionally, no patient exhibited respiratory depression or required supplemental oxygen post-PACU discharge.

## Discussion

This study evaluated the safety and effectiveness of a single intraoperative dose of methadone in bariatric surgery, demonstrating its ability to lower postoperative pain scores.

The primary outcomes measured were pain levels using the VAS at 10 minutes post-arrival in the PACU and at intervals of six, 12, and 24 hours post-surgery. The MG exhibited lower average pain scores and a more significant decrease in pain over time compared to the CG. In a study comparing methadone 0.1 mg/kg to morphine 0.1 mg/kg in patients after video laparoscopic gastroplasty, the MG had lower average pain scores. Patients receiving a single dose of methadone experienced better pain management compared to those who received morphine [2].

Traditionally, opioids are linked to adverse effects such as nausea, vomiting, respiratory depression, and excessive sedation [2,7,11]. Consequently, our secondary outcomes included the incidence of nausea and vomiting, the need for supplemental oxygen after PACU discharge, respiratory depression, and opioid consumption during the postoperative period. There was no significant difference between groups in terms of rescue morphine use within the first 24 hours, except during the PACU period. These results partially align with a prospective randomized, double-blind, controlled clinical trial that found reduced opioid morphine consumption during the PACU period but also until three days postoperative in patients allocated to the methadone group [12]. 

Methadone's high analgesic potential is attributed to its complex pharmacodynamics. It acts as a strong μ-opioid receptor (MOR) agonist, blocks the NMDA receptor by antagonizing glutamate, and inhibits the reuptake of serotonin and noradrenaline in the central nervous system [7]. Consequently, even low doses of methadone, starting at 10 mg, can provide effective analgesia [5]. 

The incidences of nausea and vomiting, need for supplementary oxygen post-PACU discharge and respiratory depression were similar between groups. It is important to note that methadone plasma levels of around 100 ng/ml (equivalent to a single dose above 30 mg) are necessary to cause significant respiratory depression [5]. In the present study, a single dose of 10 mg of methadone was administered, corresponding roughly to 0.1 mg/kg. A lower incidence of nausea and vomiting was found in the methadone group compared with the morphine and fentanyl groups in other studies [2,7]. 

Neuraxial anesthesia, such as epidural or intrathecal, is considered the "gold standard" for managing postoperative pain in abdominal surgeries. However, it carries risks of complications like infections, neurological injuries, transient neurological radiculopathy, and vertebral canal hematoma. Regional anesthesia, specifically a wall block, can provide effective and long-lasting postoperative pain relief but requires specialized training and is not suitable for all surgical and patient conditions. Obesity, for instance, can pose technical challenges for regional blocks, even with ultrasound guidance [4,13,14].

The results are limited because the evaluation period did not exceed 24 hours, as patients were discharged from the hospital approximately 24 hours after the surgical procedure. Additionally, our study only included patients who underwent laparoscopic gastroplasty, requiring more data to extrapolate to other procedures.

## Conclusions

Our findings suggest that a single dose of intravenous methadone during laparoscopic bariatric surgery is safe and leads to improved postoperative pain management, resulting in lower pain scores and reduced need for additional opioid medication during the PACU period. The 10 mg dose was found to be safe, with no occurrences of respiratory depression or need for supplemental oxygen. Therefore, methadone can be considered an effective strategy for controlling postoperative pain, reducing the need for rescue opioids, and minimizing the incidence of their adverse effects.
